# SPIRITUALITY AND RELIGIOSITY IN BRAZILIAN AND AMERICAN OLDER ADULTS, CHARACTERISTICS AND NURSING CARE APPROACH

**DOI:** 10.1093/geroni/igad104.0928

**Published:** 2023-12-21

**Authors:** Angelo Jose Bós, Amelia Gallagher

## Abstract

This symposium aims to understand two aspects of spirituality and religiosity: the importance of religiosity for older adults, in a population-based study, and a proposal approach for nursing care in cancer patients: Lift the Spirit approach. In the Brazilian study, we will show that religiosity is significantly associated with sex, race, level of education, perceived health status and non-transmittable chronic diseases, including cancer. The results evidence the important role of religious faith in the Brazilian older adult’s health. We concluded that religion and spiritual approaches should always be considered for older adults. In the American study, we will show that preliminary findings suggested Lift the Spirit was feasible, acceptable, and positively impacted knowledge, skills, and self-efficacy. The innovative combination of synchronous and asynchronous online delivery of the intervention and data collection methods has implications for research, education, and clinical practice. Lift the Spirit delivered on an entirely online format is widely accessible and convenient to researchers, educators, and practitioners, as well as participants and students. Equipping nurses to conduct spiritual histories through an intervention like Lift the Spirit is crucial to overcoming a significant barrier to providing spiritual care.

